# Knowledge, perceptions and practices regarding schistosomiasis among women living in a highly endemic rural district in Zimbabwe: implications on infections among preschool-aged children

**DOI:** 10.1186/s13071-019-3668-4

**Published:** 2019-09-23

**Authors:** Masceline Jenipher Mutsaka-Makuvaza, Zvifadzo Matsena-Zingoni, Cremance Tshuma, Agnes Katsidzira, Bonnie Webster, Xiao-Nong Zhou, Nicholas Midzi

**Affiliations:** 1 Department of Medical Microbiology, College of Health Sciences, University of Zimbabwe, P. O. Box A178, Avondale, Harare, Zimbabwe; 2 National Institute of Health Research, Ministry of Health and Child Care, P.O. Box CY573, Causeway, Harare, Zimbabwe; 3 Division of Epidemiology and Biostatistics, School of Public Health, Faculty of Health Sciences, University of Witwatersrand, 27 St Andrews’ Road, Parktown, Johannesburg, 2193 South Africa; 4 Mashonaland Central Provincial Health Office, Ministry of Health and Child Care, Bindura, Mashonaland Central Zimbabwe; 5 Harare Central Hospital, P.O Box ST 14, Southerton, Harare, Zimbabwe; 6 Department of Life Sciences, Natural History Museum, 14 Cromwell Road, London, SW7 5BD UK; 7 National Institute of Parasitic Diseases, Chinese Centre for Disease Control and Prevention, Shanghai, 200025 China

**Keywords:** Schistosomiasis, Socio-demographic knowledge, Perceptions, Practices, PSAC, Women

## Abstract

**Background:**

Schistosomiasis primarily affects poor and neglected communities due to their lack of safe water and sanitation facilities. In an effort to improve intervention strategies, the present study investigated the association of socio-demographic characteristics of women with their existing knowledge, perceptions and practices (KPP) in five urogenital schistosomiasis endemic rural communities in Zimbabwe.

**Methods:**

In February 2016, a cross sectional study was conducted in which 426 women in rural Madziwa area, Shamva District were interviewed using a pretested structured questionnaire seeking their KPP and socio-demographic characteristics. Logistic regression analysis was performed to identify socio-demographic factors associated with the KPP variables.

**Results:**

Among the 426 participants, 93.7% knew about schistosomiasis, while 97.7 and 87.5% understood the disease transmission and methods for prevention, respectively. A significantly higher percentage of women aged ≥ 30 years compared to those < 30 years indicated that infertility is a complication of untreated chronic schistosomiasis (OR: 1.7, 95% CI: 0.9–3.0). Compared to women who had no history of infection, those who had been infected before were more likely to think that they were currently infected (OR: 3.7, 95% CI: 2.4–6.0). Bathing in unsafe water sources was more common in non-apostolic compared to apostolic followers (OR: 2.1, 95% CI: 1.2–3.7). Sole use of unsafe water for domestic purposes was significantly higher in uneducated women compared to the educated (OR: 1.8, 95% CI: 1.0–3.1). Compared to women of the Chakondora community, those in Chihuri, Nduna and Kaziro were more likely to know that dysuria is a symptom of schistosomiasis while those in Chihuri were also likely to allow young children to perform water contact activities (OR: 2.9, 95% CI: 1.5–5.5).

**Conclusions:**

Despite the high level of schistosomiasis awareness, some women had inadequate knowledge about the mode of transmission and preventive measures for schistosomiasis. Socio-demographic characteristics were associated with the KPP of women. Thus, disease control efforts should consider socio-demographic factors, which may influence the knowledge, perceptions and practices of occupants in a given setting.

## Background

Schistosomiasis is a neglected tropical parasitic disease [1], particularly found among people living in communities that are generally poor with inadequate or no safe water and sanitary facilities [2, 3]. Approximately 90% of global schistosomiasis cases occur in the sub-Saharan Africa [2], where 224 million people are affected [4]. In Zimbabwe, schistosomiasis occurs in 91% of all the districts with an overall mean prevalence of 22.7% [5].

Recognising the public health impact of schistosomiasis [6–9], the 54th World Health Assembly (WHA) encouraged all endemic states to scale up mass drug administration (MDA) earmarking school-aged children (SAC) as the prime target group [10]. The recent 65th WHA schistosomiasis elimination resolution has resulted in a shift of focus from MDA, not only in SAC but also to include all high-risk groups [11]. Besides recognizing other high-risk groups such as preschool-aged children (PSAC) and women [12–18], a number of obstacles are limiting the scaling up of schistosomiasis control activities in a number of endemic countries. Efforts to treat all high-risk age groups are hindered by limited provision of praziquantel and lack of an appropriate paediatric formulation for young children [19]. Other challenges include lack of political commitment and health education to improve the understanding of the public health importance of schistosomiasis in addition to improving awareness of disease control and elimination interventions [9]. A compelling rationale for health education in any setting takes into account knowledge, perceptions and practices (KPP) of the target population. Lack of knowledge and wrong perceptions may result in people unknowingly contaminating water bodies or using unprotected water sources [20]. Despite the fact that women also perform most of the water contact activities within the family, putting them at high risk of infection, previous studies in Zimbabwe seeking knowledge and water contact behaviour have focused on SAC who are considered to be the most high risk group for infection [21, 22]. Furthermore, as caregivers of PSAC, women play a major role in influencing the water contact exposure and activities of young children [12, 23–28]. We recently reported the relationship between KPP of women caregivers and schistosomiasis infection in PSAC [29]. However, some studies have shown that the KPP of individuals regarding schistosomiasis are influenced by socio-demographic characteristics such as age, sex, occupation, level of education and religion [30–33]. In addition, low socioeconomic status results in the lack of access to safe water and improved sanitation coupled with poor hygiene practices. These localised factors lead to small-scale spatial heterogeneity of schistosomiasis. In Zimbabwe, there is a dearth of information on the association of KPP and socio-demographic characteristics of women in endemic communities. Such data are important for identifying, designing, and implementing effective community-based control interventions. Within this context, this study evaluated the schistosomiasis KPP of women and their association with the women’s socio-demographic factors in an endemic rural area in Shamva District, Zimbabwe. It is hoped that the findings from this investigation will provide preliminary insights into female related KPP and socio-demographic characteristics in Zimbabwe.

## Methods

### Study area

The study was conducted in the rural area of Madziwa, Shamva District, Mashonaland Central Province, Zimbabwe. Shamva District was selected based on its recorded high prevalence of schistosomiasis in SAC (62%) [5]. The area has been previously described [34] and is located approximately 55 km from Bindura, the nearest town with a provincial hospital. One big river (Mupfure) and three small rivers (Nyamaruru, Nyarukunda and Kamoyo) and a stream (Zvisokwe) drain the area serving as water sources for most household and farming activities. A significant number of families depend on vegetable and tobacco farming as a source of income.

### Study design and population

The study was cross-sectional in design and was conducted within a major study investigating host-schistosome interactions: Disease burden in children aged five years and below, mothers and compliance during a one year schistosomiasis control programme in a district described as highly endemic in Zimbabwe. The questionnaire was administered to women who had brought their children to the urine collection point, and included in the study as described by Mutsaka-Makuvaza et al. [29].

### Sample size

In estimating the sample size of the study population, the previously reported prevalence of 62% in Shamva District was used [5]. Also considered was the assumption that the prevalence of schistosomiasis in school-aged children (SAC) is a proxy of the surrounding population age groups [8]. The required sample size of 363 women was calculated using Dobson’s formula as follows:where *Z* is the *Z*-value for the 95% confidence interval, that is alpha = 5% (*z* = 1.96), *p* is the proportion/prevalence of the outcome to be investigated (*p* = 0.62), *q* = 1 − *p* = 0.38, *d* is the precision for the given confidence interval expected expressed as a decimal (*d* = 0.05) and *n* = 363.

Due to the overwhelming response from the women, 426 participated in the questionnaire survey.

### Interview using questionnaire

The questionnaire was developed and administered as described by Mutsaka-Makuvaza et al. [29]. The validated Shona survey questionnaire sourcing for socio-demographic characteristics, and KPP related to schistosomiasis infection was used to collect data from all women attending the survey. Variables such as age of the participant, religion, employment status, education status, perceptions regarding high risk groups, sanitary practices and water contact behaviour were captured. Additional information on the knowledge of current and past occurrences, transmission of urogenital schistosomiasis, symptoms, and prevention methods of infection were captured from the women as previously described [29].

### Data analysis

The collected data were double entered into excel and analysed using STATA v.15.2 (Stata Corp, College Station, TX, USA). The data were summarised using descriptive statistics, including percentages and frequency values. We also performed logistic regression analysis to identify socio-demographic characteristics associated with the KPP variables among the participants. Association between KPP variables and all communities was assessed using the Chi-square test. Odds ratios (OR) and 95% confidence intervals (95% CI) were generated for all variables. Univariate and multivariate logistic regression analysis were performed with bathing in unsafe water as the dependent variable while women’s community of residence, age, education status, employment status, religion and history of infection as the independent variables. For the inclusion of variables in the multivariate logistic regression, we set a liberal *P*-value at 0.1. Statistical significance was set at 5% in all analyses.

## Results

### Socio-demographic characteristics of the study participants

All 426 women were interviewed. The age profiles for the respondents are described in Table 1. There were more educated women (58.2%) than uneducated ones (41.8%). African apostolic sect followers (85.2%) dominated in the area (Table 1). While the overall number of women who used toilets was 362 (85.0%), 343 had toilets at home but 4 (1.2%) of them did not use the toilets. Amongst the 362 women who used toilets, 23 (6.4%), participants used their neighbours’ toilets. Of the 61 women who had *S. haematobium* infections, 11 (18.0%) had no toilet.

**Table 1:** Socio-demographic characteristics of study participants from Madziwa area, Shamva district, 2017

Variable	Characteristic	Frequency	Percentage
Adult age group (years)	≤ 20	23	5.4
	21–25	74	17.4
	26–30	98	23.0
	31–35	97	22.8
	36–40	73	17.1
	> 40	61	14.3
Socioeconomic status			
Education^a^	Not educated	178	41.8
	Educated	248	58.2
Employment^b^	Employed	84	19.7
	Farmer	73	17.1
	ECD teachers	2	0.5
	Hairdressers	2	0.5
	Village health workers	3	0.7
	Youth officers	1	0.2
	Domestic workers	2	0.5
	Tailors	1	0.2
	Not employed	342	80.3
Religion	Traditional	1	0.2
	Muslims	1	0.2
	Christians	424	99.5
	Mainline church^d^	10	2.3
	Protestants^e^	25	5.9
	Pentecostal^f^	26	6.1
	African apostolic^g^	363	85.2
Domestic source of water^c^	Tap	0	0
	Borehole	265	62.2
	Dam	9	2.1
	River	96	22.5
	Well	125	29.3
Toilet	Those with a toilet at home	343	80.5
	Those who use the toilet	362	85.0

*Notes*: The table describes the age of the women, community of origin, education level, employment status, religious beliefs, their domestic water source, presence and use of toilet

^a^All women who went to school only up to primary level or below were considered as not educated. Participants were considered to be educated if they attained secondary level of education

^b^All women who had some form of income generating activity were considered employed. This included the teachers, hairdressers, village health workers, youth officers, small-scale farmers, domestic workers and tailors. Women were considered not employed if they had no income generating activity

^c^Multiple responses were considered

^d^Roman Catholic

^e^Reformed church in Zimbabwe, Methodist and Lutheran

^f^ZAOGA, Apostolic Faith Mission in Zimbabwe, Endtime message and New Revelation

^g^Johane Masowe, Madzibaba and Nguotsvuku

### Knowledge of women about schistosomiasis

Table 2 describes women’s knowledge about schistosomiasis symptoms, transmission and prevention. The majority of women knew about schistosomiasis 399 (93.7%) and its treatment 395 (99.0%). Amongst the participants, 29.8% thought that they were currently infected. Of the 97.7% respondents who knew at least one method of schistosomiasis transmission, 92.7% knew that schistosomiasis transmission occurs through contact with contaminated water. Among the 399 participants who knew about schistosomiasis, 92.0% knew at least one symptom of schistosomiasis infection, 73.4% indicated haematuria as a symptom of schistosomiasis, while 3.8% indicated that they did not know the symptoms for infection. The participants showed a relatively low level of knowledge of the complications associated with chronic schistosomiasis (57.1%). While 98.3% of the participants were aware of the annual national mass drug administration (MDA) programme (mass praziquantel treatment), only 25.8% thought that anti-schistosomal treatment was a prevention and control method. Of the respondents, 44.4% believed that avoiding unsafe water bodies prevents and controls schistosomiasis transmission.

**Table 2:** Women’s knowledge about schistosomiasis symptoms, transmission and prevention

Variable	Characteristic	Frequency	Percentage
Knowledge of schistosomiasis infection and treatment			
	Women who knew that bilharzia is a disease	399	93.7
	Women who had bilharzia infection before	205	51.4
	Women who thought they were currently infected	127	29.8
	Those who knew praziquantel as a drug for bilharzia treatment	395	99.0
	Those who stated health centre as a place of bilharzia treatment	395	99.0
What are the modes of bilharzia transmission?^a^			
	Those who stated at least one transmission mode^b^	390	97.7
	Contact with contaminated water	370	92.7
	Drinking dirty water	5	1.3
	Contact with contaminated water and entering toilets barefooted	7	1.8
	Contact with contaminated water and drinking dirty water	15	3.8
	Don’t know	2	0.5
What are the signs and symptoms of bilharzia infection?^a^			
	Those who stated at least one symptom	367	92.0
	Haematuria	293	73.4
	Weight loss	98	24.6
	General body weakness	51	12.8
	Headache	4	1.0
	Dysuria	39	9.8
	Poor school performance	5	1.3
	Abdominal pain	26	76.5
	Genital itchiness in women	8	2.0
	Nausea	35	8.8
	Infertility	4	1.0
	Recurrent illness	7	1.8
	I don’t know	15	3.8
What are the complications of untreated chronic bilharzia infection?^a^			
	Those who stated at least one complication	228	57.1
	Infertility	170	42.6
	Mental disturbance	54	13.5
	Death	31	7.8
	I don’t know	16	4.0
What are the prevention and control methods of bilharzia?^a^			
	Those who stated at least one prevention and control method	349	87.5
	Taking anti-schistosomal medicines	103	25.8
	Avoiding use of unprotected water bodies	177	44.4
	Health education	36	9.1
	Provision of WASH facilities	62	15.6
Are you aware of the current annual national mass drug administration programme?			
	Yes	392	98.3

*Notes*: The table shows percentages of participants with knowledge on schistosomiasis infection, treatment, modes of transmission, symptoms of infection and complications of untreated chronic infections. Awareness of the current ongoing annual national mass drug administration, the prevention and control methods of schistosomiasis as mentioned by respondents are also described

^a^Multiple responses were considered

^b^Those who mention the transmission mode regardless of other incorrect transmission mode responses

*Abbreviation*: WASH, water, sanitation and hygiene

### Perceptions and practices of women to schistosomiasis

Results on perceptions and practices of the participants towards schistosomiasis are described in Table 3. The majority of women reported that SAC were the most at risk group (76.9%) and regarded MDA as an important programme (99.8%) that they were willing to be part of (99.0%). Amongst the respondents, 66.7% reported that they exposed their children aged 5 and below to unsafe water sources, 43.4% allowed the children to play in shallow water and 14.1% placed the children in water-filled basins at all times during other water activities, particularly laundry. Meanwhile, 162 (38.0%) of the participants indicated that they used unprotected water sources (rivers and dams) for bathing. Among the participants, 77.5% had vegetable gardens, while 93.0% and 32.4% of these used unsafe river water and allowed their PSAC to assist with watering the garden, respectively.

**Table 3:** Perceptions and practice of women in rural Madziwa to schistosomiasis

Question	Responses	Frequency	Percentage
Risky perceptions			
Which population is most likely infected?^a^	Children aged 5 years and below	199	49.9
	School-aged children	307	76.9
	Adult women	79	19.8
	Adult men	54	13.5
	Girls	67	16.8
	Boys	74	18.6
Is the national annual MDA programme important?	Yes	398	99.8
	No	1	0.3
Are you willing to participate in the annual MDA programme?	Yes	395	99.0
	No	4	1.0
Practices			
Where do you do your laundry?	River	309	72.5
	Dam	28	6.6
	Garden well	16	3.8
	Borehole	13	3.1
	Well at home	60	14.1
What time do you do laundry?	Morning	240	56.3
	Afternoon	155	36.4
	Late afternoon	31	7.3
Do you take your PSAC when going for laundry?	Yes	284	66.7
	No	116	27.2
	Sometimes	26	6.1
Where does your PSAC play when you are washing?	In shallow water	185	43.4
	In water basin filled with water	60	14.1
	Outside water	128	30.1
	Do not take the child along to the river for washing	116	12.4
Where do you go for bathing?	River	157	36.9
	Dam	5	1.2
	Garden well	7	1.6
	Borehole	15	3.5
	Home using well water	242	56.8
What time do you go for bathing?	Morning	22	5.2
	Afternoon	87	20.4
	Late afternoon	317	74.4
Do you take your PSAC when going for bathing?	Yes	220	51.6
	No	169	39.7
	Sometimes	37	8.7
Where does your PSAC play when you are bathing?	Playing in water bathing also	158	61.5
	Playing in shallow water	54	21.0
	In water basin filled with water	45	17.5
Do you have a vegetable garden?	Yes	330	77.5
	No	96	22.5
Where do you fetch water for gardening?	River	307	93.0
	Garden well	23	7.0
	Borehole	0	0
	Tap	0	0
Does the PSAC assist with gardening?	Yes	107	32.4
	No	201	60.9
	Sometimes	22	6.7
Do you discuss bilharzia with children at home?	Yes	374	93.7
	No	25	6.3

*Notes*: The table describes the details of the perceptions of women regarding groups at risk of infection. It also outlines the different water sources, water contact practices of women such as washing, bathing and gardening

^a^Multiple responses were considered

*Abbreviation*: PSAC, preschool-aged child

### Association of women’s knowledge, perceptions and practices on schistosomiasis with their age and religion

Table 4 shows that women aged ≥ 30 were more likely to know that infertility is a complication of untreated chronic schistosomiasis (OR: 1.6, 95% CI: 1.1–2.4) and were also more likely to discuss schistosomiasis with their children at home (OR: 4.5, 95% CI: 1.7–12.2) than women aged less than 30 years. The apostolic followers were less likely to know that avoiding unprotected water bodies is a schistosomiasis prevention method compared to non-apostolic followers (OR: 0.6, 95% CI: 0.3–1.0). However, they were more likely to use safe water for bathing (OR: 2.1, 95% CI: 1.2–3.7) but less likely to have and to use a toilet at their homes compared to the non-apostolic followers (OR: 0.4, 95% CI: 0.2–0.9 and OR: 0.2, 95% CI: 0.1–0.8, respectively).

**Table 4:** Association of knowledge of women about schistosomiasis with their age and religion

Variable	Age (years)				Religion			
	< 30 *n* (%)	≥ 30 *n* (%)	OR	95% CI	Non-apostolic *n* (%)	Apostolic *n* (%)	OR	95% CI
Knowledge of bilharzia								
Woman thinks she is currently infected	61 (31.1)	61 (30.1)	1.0	0.6–1.5	20 (32.3)	102 (30.3)	0.9	0.5–1.6
What are the signs and symptoms of bilharzia infection?^a^								
Haematuria	138 (70.4)	155 (76.4)	1.4	0.9–2.1	45 (72.6)	248 (73.6)	1.1	0.6–1.9
Weight loss	46 (23.5)	52 (25.6)	1.1	0.7–1.8	9 (14.5)	89 (26.4)	2.1	1.0–4.5**
Dysuria	19 (9.7)	20 (9.9)	1.0	0.5–2.0	7 (11.3)	32 (9.5)	0.8	0.3–2.0
Abdominal pain	9 (4.6)	17 (8.4)	1.9	0.8–4.4	3 (4.8)	23 (6.8)	1.4	0.4–5.0
Genital itchiness in women	2 (1.0)	6 (3.0)	2.9	0.6–14.7	1 (1.6)	7 (2.1)	1.3	0.2–10.7
I don’t know	8 (4.1)	7 (3.5)	0.8	0.3–2.4	1 (1.6)	14 (4.2)	2.6	0.3–20.5
What are the complications of untreated chronic bilharzia infection?^a^								
Infertility	72 (36.7)	98 (48.3)	1.6	1.1–2.4*	20 (32.3)	150 (44.5)	1.7	0.9–3.0
Mental disturbance	21 (10.7)	33 (16.3)	1.6	0.9–2.9	10 (16.1)	44 (13.1)	0.8	0.4–1.6
Death	14 (7.1)	17 (8.4)	1.2	0.6–2.5	9 (14.5)	22 (6.5)	0.4	0.2–0.9*
I don’t know	8 (4.1)	8 (3.9)	1.0	0.4–2.6	5 (8.1)	11 (3.3)	0.4	0.1–1.1
What are the prevention and control methods of bilharzia?^a^								
Taking anti-schistosomal medicines	55 (28.1)	48 (23.7)	0.8	0.5–1.2	13 (21.0)	90 (26.7)	1.4	0.7–2.7
Avoiding use of unprotected water bodies	86 (43.9)	91 (44.8)	1.0	0.7–1.5	35 (56.5)	142 (42.1)	0.6	0.3–1.0*
Health education	19 (9.7)	17 (8.4)	0.8	0.4–1.7	4 (6.5)	32 (9.5)	1.5	0.5–4.5
Provision of WASH facilities	29 (14.8)	33 (16.3)	1.1	0.7–1.9	11 (18.0)	51 (15.1)	0.8	0.4–1.7
Risky perceptions^a^								
School-aged children most likely infected	149 (76.0)	158 (77.8)	1.1	0.7–1.8	45 (72.6)	262 (77.7)	1.3	0.7–2.4
Children aged ≤ 5 years most likely infected	102 (52.0)	97 (47.8)	0.8	0.6–1.2	35 (56.5)	164 (48.7)	0.7	0.4–1.3
Adult women most likely infected	33 (16.8)	46 (22.7)	1.4	0.9–2.4	10 (16.1)	69 (20.5)	1.3	0.6–2.8
Adult men most likely infected	20 (10.2)	34 (16.8)	1.8	1.0–3.2**	5 (8.1)	49 (14.5)	1.9	0.7–5.1
Practices^a^								
Use unsafe water for domestic purposes^b^	29 (13.7)	30 (14.0)	1.0	0.6–1.8	8 (12.7)	51 (14.1)	1.1	0.5–2.5
Use unsafe water for laundry^b^	179 (84.4)	174 (81.3)	1.2	0.8–2.1	52 (82.5)	301 (82.9)	1.0	0.5–2.0
Bath in unsafe water^b^	84 (39.6)	85 (39.7)	1.0	0.7–1.5	35 (55.6)	134 (36.9)	2.1	1.2–3.7*
Allow PSAC to play in unsafe water while they are bathing	118 (55.7)	127 (59.4)	0.9	0.6–1.3	38 (60.3)	207 (57.0)	1.1	0.7–2.0
Bath PSAC using boiled water	198 (93.4)	198 (92.5)	0.9	0.4–1.8	58 (92.1)	338 (93.1)	1.2	0.4–3.2
Allow PSAC to help water the garden	56 (26.4)	68 (31.8)	1.3	0.9–2.0	24 (38.1)	100 (27.6)	0.6	0.4–1.1
Have a toilet at home	170 (80.2)	173 (80.8)	1.0	0.6–1.7	57 (90.5)	286 (78.8)	0.4	0.2–0.9*
Use of a toilet for excreta disposal	180 (84.9)	182 (85.1)	1.0	0.6–1.7	60 (95.2)	302 (83.2)	0.2	0.1–0.8*
Discussion of bilharzia at home	176 (89.8)	198 (97.5)	4.5	1.7–12.2*	58 (93.6)	316 (93.8)	1.0	0.3–3.1

^a^Multiple responses were considered

^b^Those who rely solely on unsafe water for the indicated water contact activities

* Significant association (*P* < 0.05); ** Borderline significance (*P* = 0.05)

*Abbreviations*: OR, odds ratio; 95% CI, 95% confidence interval; PSAC, preschool-aged children; WASH, water, sanitation and hygiene

### Association of women’s knowledge, perceptions and practices on schistosomiasis with their education and employment status

Table 5 shows that those who were educated were 40% less likely to rely solely on unsafe water sources for domestic purposes (OR: 0.6, 95% CI: 0.3–1.0) but more likely to perceive women as a high-risk group (OR: 1.9, 95% CI: 1.1–3.2) compared to the uneducated women. The odds of knowing that haematuria is a symptom of schistosomiasis was higher in educated participants (OR: 1.7, 95% CI: 1.1–2.6) compared to the uneducated women. Table 5 also shows that the odds of perceiving that SAC are a high risk group was two times lower in unemployed women compared to employed women (OR: 2.1, 95% CI: 1.1–4.2). Compared to the employed women, unemployed women were less likely to know that treatment is a schistosomiasis control measure (OR: 2.5, 95% CI: 1.5–4.2) and more likely to allow PSAC to play in unsafe water when bathing (OR: 2.0, 95% CI: 1.2–3.2).

**Table 5:** Association of knowledge, perceptions and practices of the women regarding schistosomiasis with their education and employment status (*n* = 426)

Variable	Education				Employment			
	Not educated *n* (%)	Educated *n* (%)	OR	95% CI	Not employed *n* (%)	Employed *n* (%)	OR	95% CI
Knowledge of bilharzia								
Woman thinks she is currently infected	52 (31.9)	70 (29.6)	0.9	0.6–1.4	103 (32.3)	19 (23.8)	0.7	0.4–1.2
What are the signs and symptoms of bilharzia infection?^a^								
Haematuria	110 (67.5)	183 (77.5)	1.7	1.1–2.6*	231 (72.4)	62 (77.5)	1.3	0.7–2.3
Weight loss	54 (33.1)	44 (18.6)	0.5	0.3–0.7*	76 (23.8)	22 (27.5)	1.2	0.7–2.1
Dysuria	14 (8.6)	25 (10.6)	1.3	0.6–2.5	35 (11.0)	4 (5.0)	0.4	0.1–1.2
Abdominal pain	11 (6.8)	15 (6.4)	0.9	0.4–2.1	22 (6.9)	4 (5.0)	0.7	0.2–2.1
Genital itchiness in women	5 (3.1)	3 (1.3)	0.4	0.1–1.7	7 (2.2)	1 (1.3)	0.6	0.1–4.6
I don’t know	2 (1.2)	13 (5.5)	4.7	1.0–21.1*	15 (4.7)	0 (0)	–	–
What are the complications of untreated chronic bilharzia infection?^a^								
Infertility	61 (37.4)	109 (46.2)	1.4	1.0–2.2	131 (41.1)	39 (48.8)	1.4	0.8–2.2
Mental disturbance	22 (13.5)	32 (13.6)	1.0	0.6–1.8	48 (15.1)	6 (7.5)	0.5	0.2–1.1
Death	11 (6.8)	20 (8.5)	1.3	0.6–2.7	27 (8.5)	4 (5.0)	0.6	0.2–1.7
I don’t know	5 (3.1)	11 (4.7)	1.5	0.5–4.5	16 (5.0)	0 (0)	–	–
What are the prevention and control methods of bilharzia?^a^								
Taking anti-schistosomal medicines	47 (28.8)	56 (23.7)	0.8	0.5–1.2	70 (21.9)	33 (41.3)	2.5	1.5–4.2*
Avoiding use of unprotected water bodies	73 (44.8)	104 (44.1)	1.0	0.7–1.5	147 (46.1)	30 (37.5)	0.7	0.4–1.2
Health education	14 (8.6)	22 (9.4)	1.1	0.5–2.2	34 (10.7)	2 (2.5)	0.2	0.1–0.9*
Provision of WASH facilities	24 (14.7)	38 (16.2)	1.1	0.6–1.9	54 (16.9)	8 (10.1)	0.6	0.3–1.2
Risky perceptions^a^								
School-aged children most likely infected	124 (76.1)	183 (77.5)	1.1	0.7–1.7	238 (74.6)	69 (86.3)	2.1	1.1–4.2*
Children aged ≤ 5 years most likely infected	78 (47.9)	121 (51.3)	1.1	0.8–1.7	152 (47.7)	47 (58.8)	1.6	1.0–2.6
Adult women most likely infected	23 (14.1)	56 (23.7)	1.9	1.1–3.2*	62 (19.4)	17 (21.3)	1.1	0.6–2.0
Adult men most likely infected	13 (8.0)	41 (17.4)	2.4	1.3–4.7*	43 (13.5)	11 (13.8)	1.0	0.5–2.1
Practices^a^								
Use unsafe water for domestic purposes^b^	32 (18.0)	27 (10.9)	0.6	0.3–1.0*	48 (14.0)	11 (13.1)	0.9	0.5– 1.9
Use unsafe water for laundry^b^	151 (84.8)	202 (81.5)	1.3	0.8–2.1	284 (83.0)	69 (82.1)	1.1	0.6–2.0
Bath in unsafe water^b^	70 (39.3)	99 (39.9)	1.0	0.7–1.4	139 (40.6)	30 (35.7)	1.2	0.8–2.0
Allow PSAC to play in unsafe water while they are bathing	99 (55.6)	146 (58.9)	0.9	0.6–1.3	208 (60.8)	37 (44.1)	2.0	1.2–3.2*
Bath PSAC using boiled water	171 (96.1)	225 (90.7)	0.4	0.2–1.0*	315 (92.1)	81 (96.4)	2.3	0.7–7.8
Allow PSAC to help water the garden	50 (28.1)	74 (29.8)	1.1	0.7–1.7	101 (29.5)	23 (27.4)	0.9	0.5–1.5
Have a toilet at home	142 (79.8)	201 (81.1)	1.1	0.7–1.8	272 (79.5)	71 (84.5)	1.4	0.7–2.7
Use of a toilet for excreta disposal	148 (83.2)	214 (86.3)	1.3	0.7–2.2	290 (84.8)	72 (85.7)	1.1	0.5–2.1
Discussion of bilharzia at home	154 (94.5)	220 (93.2)	0.8	0.3–1.9	299 (93.7)	75 (93.8)	1.0	0.4–2.8

^a^Multiple responses were considered

^b^Those who rely solely on unsafe water for the indicated water contact activities

* Significant association of *P* < 0.05; ** Borderline significance *P* = 0.05

*Abbreviations*: OR, odds ratio; 95% CI, 95% confidence interval; PSAC, preschool-aged children; WASH, water, sanitation and hygiene

### Association of women’s knowledge, perceptions and practices about schistosomiasis with community of residence

Table 6 and Additional file 1: Table S1 show that there were significant associations of knowledge, perceptions, and practices of the women regarding schistosomiasis with their community of residence. Compared to women residing in Chakondora community, the odds of knowing that dysuria is a symptom of schistosomiasis was significantly higher in Chihuri (OR: 8.2, 95% CI: 1.8–37.3), Kaziro (OR: 1.7, 95% CI: 1.5–34.8) and Nduna (OR: 5.9, 95% CI: 1.1–31.6). Meanwhile, residence of Mupfure were more likely to know that haematuria is a symptom of schistosomiasis (OR: 3.7, 95% CI: 1.8–7.4) and they relied solely on unsafe water for domestic purposes (OR: 2.6, 95% CI: 1.1–5.9) compared to residence of Chakondora. Participants in Chihuri were more likely to allow their children to assist in water contact activities, while in Nduna they were less likely to have and to use the toilet compared to those from Chakondora.

**Table 6:** Association of knowledge, perceptions and practices of women regarding schistosomiasis with their community of residence

Variable	Community							
	Chihuri		Kaziro		Mupfure		Nduna	
	OR	95% CI	OR	95% CI	OR	95% CI	OR	95% CI
Knowledge of bilharzia								
Caregiver thinks she is currently infected	1.5	0.8–3.1	1.2	0.6–2.7	2.4	1.3–4.5*	0.7	0.3–1.9
What are the signs and symptoms of bilharzia infection?^a^								
Haematuria	0.8	0.4–1.5	0.9	0.5–1.9	3.7	1.8–7.4*	1.0	0.4–2.1
Weight loss	0.3	0.1–0.5*	0.2	0.1–0.4*	0.3	0.1–0.5*	0.6	0.3–1.3
Dysuria	8.2	1.8–37.3*	7.2	1.5–34.8*	3.0	0.6–14.4	5.9	1.1–31.6*
Abdominal pain	6.4	0.8–54.2	1.4	0.1–22.9	6.9	0.9–54.6	20.8	2.5–172.6*
Genital itchiness in women	0.7	0.1–3.7	–	–	0.3	0.0–1.8	–	–
I don’t know	0.4	0.1–1.8	–	–	0.4	0.1–1.6	0.6	0.1–3.1
What are the complications of untreated chronic bilharzia infection?^a^								
Infertility	1.2	0.6–2.1	0.2	0.1–0.4*	0.4	0.3–0.8*	0.2	0.1–0.5*
Mental disturbance	0.3	0.1–0.8*	0.8	0.3–2.0	0.7	0.3–1.5	1.2	0.4–3.0
Death	0.4	0.1–1.6	0.8	0.2–2.8	1.5	0.6–3.9	0.3	0.0–2.4
I don’t know	1.0	0.2–5.1	3	0.7–12.5	0.9	0.2–3.9	–	–
What are the prevention and control methods of bilharzia?^a^								
Taking anti-schistosomal medicines	0.1	0.1–0.3*	0.3	0.2–0.6*	0.2	0.1–0.3*	0.3	0.1–0.7*
Avoiding use of unprotected water bodies	1.2	0.6–2.1	1.0	0.5–1.9	1.3	0.7–2.2	0.6	0.3–1.4
Health education	3.6	1.1–11.6*	4.0	1.2–13.3*	1.3	0.4–4.4	0.5	0.1–4.7
Provision of WASH facilities	1.8	0.7–4.9	3.3	1.2–8.8*	2.1	0.8–5.1	2.7	0.9–8.1
Risky perceptions^a^								
School-aged children most likely infected	0.6	0.2–1.9	0.1	0.0–0.3*	0.2	0.1–0.6*	0.1	0.0–0.2*
Children aged ≤ 5 years most likely infected	0.3	0.1–0.6*	3.6	1.8–7.5*	2.0	1.1–3.4*	1.1	0.5–2.3
Adult women most likely infected	0.2	0.0–0.6*	1.5	0.7–3.4	2.0	1.0–3.9*	0.2	0.0–1.0**
Adult men most likely infected	0.2	0.1–0.9*	0.7	0.3–2.1	2.1	1.0–4.3	0.5	0.1–2.0
Practices^a^								
Use unsafe water for domestic purposes^b^	2.1	0.8–5.2	0.8	0.2–2.3	2.6	1.1–5.9*	1.0	0.3–3.4
Use unsafe water for laundry^b^	0.6	0.3–1.4	1.4	0.7–3.0	0.7	0.3–1.3	0.2	0.0–0.8*
Bath in unsafe water^b^	0.5	0.3–1.0*	0.4	0.2–0.8*	0.3	0.2–0.6	0.2	0.1–0.5*
Allow PSAC to play in unsafe water while they are bathing	0.4	0.2–0.8*	0.9	0.5–1.8	0.5	0.3–0.8	1.5	0.7–3.0
Bath PSAC using boiled water	1.7	0.6–4.8	–	–	1.4	0.5–3.5	3.1	0.6–15.6
Allow PSAC to help water the garden	2.9	1.5–5.5*	1.5	0.7–3.0	1.4	0.8–2.7	0.3	0.1–1.0**
Have a toilet at home	1.5	0.7–3.5	1.5	0.6–3.8	0.7	0.4–1.4	0.4	0.2–0.9*
Use of a toilet for excreta disposal	1.9	0.6–5.8	0.8	0.3–2.2	0.5	0.2–1.0	0.2	0.1–0.6*
Discussion of bilharzia at home	2.1	0.4–11.5	0.4	0.1–1.4	0.8	0.2–2.3	0.6	0.1–2.9

*Note*: Chakondora community was considered as the reference group

^a^Multiple responses were considered

^b^Those who rely solely on unsafe water for the indicated water contact activities

* Significant association of *P* < 0.05; ** Borderline significance *P* = 0.05

*Abbreviations*: OR, odds ratio; 95% CI, 95% confidence interval; PSAC, preschool-aged children; WASH, water, sanitation and hygiene

### Association of women’s knowledge, perceptions and practices about schistosomiasis with their history of infection

Table 7 describes the association of knowledge, perceptions and practices of the women regarding schistosomiasis with their history of infection. Compared to women with no history of infection, women who had schistosomiasis before were more likely to think they were currently infected (OR: 3.7, 95% CI: 2.4–6.0). Meanwhile, those who had been infected before were less likely to depend solely on unsafe water for bathing and laundry compared to those who had had no infection before. However, they were also more likely to allow their children to assist in watering the garden compared to those who had no infection before (OR: 1.9, 95% CI: 1.2–3.0).

**Table 7:** Association of knowledge, perceptions and practices of women regarding schistosomiasis with their history of schistosomiasis infection

Variable	Have been infected before			
	Yes *n* (%)	No *n* (%)	OR	95% CI
Knowledge of bilharzia				
Woman thinks she is currently infected	89 (43.4)	33 (17.0)	3.7	2.4–6.0*
What are the signs and symptoms of bilharzia infection?^a^				
Haematuria	155 (75.6)	138 (71.1)	1.3	0.8–2.0
Weight loss	56 (27.3)	42 (21.7)	1.4	0.9–2.2
Dysuria	22 (10.7)	17 (8.8)	1.3	0.6–2.4
Abdominal pain	13 (6.3)	13 (6.7)	0.9	0.4–2.1
Genital itchiness in women	6 (2.9)	2 (1.0)	2.9	0.6–14.6
I don’t know	6 (2.9)	9 (4.6)	0.6	0.2–1.8
What are the complications of untreated chronic bilharzia infection?^a^				
Infertility	89 (43.4)	81 (41.8)	1.1	0.8–1.6
Mental disturbance	36 (17.6)	18 (9.3)	2.1	1.1–3.8*
Death	10 (4.9)	21 (10.8)	0.4	0.2–0.9*
I don’t know	7 (3.4)	9 (4.6)	0.7	0.3–2.0
What are the prevention and control methods of bilharzia?^a^				
Taking anti-schistosomal medicines	58 (28.3)	45 (23.2)	1.3	0.8–2.1
Avoiding use of unprotected water bodies	93 (45.3)	84 (43.3)	1.1	0.7–1.6
Health education	15 (7.3)	21 (10.9)	0.6	0.3–1.3
Provision of WASH facilities	26 (12.7)	36 (18.7)	0.6	0.4–1.1
Risky perceptions^a^				
School-aged children most likely infected	148 (72.2)	159 (82.0)	0.6	0.4–0.9*
Children aged ≤ 5 years most likely infected	100 (48.8)	99 (51.0)	0.9	0.6–1.4
Adult women most likely infected	43 (21.0)	36 (18.6)	1.2	0.7–1.9
Adult men most likely infected	33 (16.1)	21 (10.8)	1.6	0.9–2.8
Practices^a^				
Use unsafe water for domestic purposes^b^	35 (17.1)	21 (10.8)	1.7	1.0–3.0
Use unsafe water for laundry^b^	178 (86.8)	154 (79.4)	0.6	0.3–1.0*
Bath in unsafe water^b^	94 (45.9)	68 (35.1)	0.6	0.4–1.0*
Allow PSAC to play in unsafe water while they are bathing	133 (64.9)	98 (50.5)	0.6	0.4–0.8*
Bath PSAC using boiled water	189 (92.0)	181 (93.3)	0.8	0.4–1.8
Allow PSAC to help water the garden	75 (36.6)	45 (23.2)	1.9	1.2–3.0*
Have a toilet at home	164 (80.0)	159 (82.0)	0.9	0.5–1.5
Use of a toilet for excreta disposal	170 (82.9)	170 (87.6)	0.7	0.4–1.2
Discussion of bilharzia at home	196 (95.6)	178 (91.8)	2.0	0.8–4.5

^a^Multiple responses were considered

^b^Those who rely solely on unsafe water for the indicated water contact activities

* Significant association of *P* < 0.05

*Abbreviations*: OR, odds ratio; 95% CI, 95% confidence interval; PSAC, preschool-aged children; WASH, water, sanitation and hygiene

### Univariate and multivariate analysis

Table 8 describes the univariate and multivariate logistic regression analysis for the location of bathing of women in relation to their socio-demographic characteristics. Univariate analysis showed that bathing in unsafe water sources was associated with the community of residence, history of infection and religion of the participant. Multivariate analysis revealed that the odds of bathing in unsafe water sources were higher in Chihuri (AOR: 0.5, 95% CI: 03–1.0), while those who had a history of infection were less likely to bath in unsafe water sources (AOR: 0.6, 95% CI: 0.4–1.0).

**Table 8:** Association of bathing place for women with their socio-demographic characteristics. Univariate and multivariate logistic regression analysis of bathing place for women in relation to their community of residence history of schistosomiasis infection, age, education status, religious belief and employment status

Variable	Category	Univariate analysis			Multivariate analysis		
		OR	95% CI	*P*-value	AOR	95% CI	*P*-value
Community of residence	Chakondora	1			1		
	Chihuri	0.5	0.2–1.0	0.044	0.5	0.3–1.0	0.047
	Kaziro	0.4	0.2–0.8	0.010	0.4	0.2–0.7	0.006
	Mupfure	0.3	0.2–0.7	0.001	0.7	0.2–0.6	0.001
	Nduna	0.2	0.1–0.5	0.001	0.3	0.2–0.7	0.006
Have been infected with schistosomiasis before	No	1			1		
	Yes	0.6	0.4–1.0	0.031	0.6	0.4–1.0	0.043
Age (years)	< 30	1					
	≥ 30	1.0	0.7–1.6	0.910			
Education status	Uneducated	1					
	Educated	1.1	0.7–1.7	0.712			
Religion	Non-apostolic	1					
	Apostolic	2.0	1.1–3.5	0.018			
Employment status	Not employed	1					
	Employed	0.8	0.4–1.4	0.352			

Reference group is marked as OR = 1

*Abbreviations*: OR, odds ratio; 95% CI, 95% confidence interval; AOR, adjusted odds ratio

## Discussion

To our knowledge, this study is the first attempt to demonstrate the association of women’s knowledge, perceptions and practices with their socio-demographic factors in relation to schistosomiasis awareness and transmission, in an endemic rural setting in Zimbabwe. The results show that the majority of the women were educated, but unemployed, knew about schistosomiasis and a significant number of them undertook their water contact activities in unsafe water sources.

The present study was implemented in an endemic area where previous schistosomiasis control programmes where conducted [16, 17] and annual MDA targeted at SAC is also ongoing. This may explain why 93.7% of the participants were aware of the disease. Almost half of the participants declared a history of infection, substantiating the endemicity of the disease in the area.

The high level of schistosomiasis awareness among women in this study is corroborated by findings from a previous study conducted in Malawi [35] in the same age group. In contrast, another study in the same country [28] reported poor knowledge of the disease among women. This calls for more studies in women from different settings to improve the benefits of targeted schistosomiasis control interventions, particularly education and increased awareness.

The present study revealed inadequate knowledge on dysuria as a symptom of urogenital schistosomiasis infection and the complications of chronic untreated schistosomiasis infection among the women. This is despite the fact that previous studies carried out in the same area focused on female genital schistosomiasis (FGS) and its complications [36–38]. Moreover, the present study was implemented in an endemic area currently undergoing MDA in SAC, which most of the respondents indicated as important and their willingness to participate if the programme was to be extended to the whole community. As such, it would be expected that most of the female community would have knowledge of the signs and symptoms of the disease. However, a considerable number of women showed confusion surrounding schistosomiasis transmission with some stating that the disease is transmitted by a combination of contact with contaminated water and also drinking dirty water. Mazigo et al. [39] also observed the same findings but from SAC. Such incorrect beliefs are due to lack of health education regarding schistosomiasis, whose provision could precede routine national MDA campaigns. However, the current MDA embedded health education campaigns carried out by the community nurses and village health workers only encourage parents to allow children to be treated. In addition, text messages are sent to individuals and posters are distributed to community health centres by the ministry. The short text messages informing the public on the dates of the MDA and indicating that treatment prevents the spread of schistosomiasis do not elaborate on the transmission, symptoms, prevention and control methods of the disease.

The study shows that there is poor knowledge about treatment for schistosomiasis and also health education as a schistosomiasis prevention and control measure. Knowledge of diseases without proper understanding of its mode of transmission, signs and symptoms, complications, prevention, and control methods is not only insufficient [20] but also less useful in control and elimination of the disease. On the other hand, while the literacy rate and the number of people with mobile phones in the area has not been established, the sending of mobile phone messages hinders the dissemination of information to those who are illiterate or individuals who do not have mobile phones. Effectiveness of the dissemination of information *via* posters is also dependent on the literacy of the targeted population. Some studies have already shown that the use of posters in dissemination of information is not effective [39, 40]. Thus, there is a need to plan comprehensive health education strategies if the 2025 schistosomiasis elimination goal is to be achieved by endemic member states [11].

Although almost half of the women included in this study had a history of schistosomiasis infection, they were more likely to use unsafe water sources, probably due to lack of adequate safe water or had lack of knowledge regarding the transmission of the disease and its prevention and control methods. Although there are available boreholes providing safe water, they are few and widely scattered such that some of the residents have to travel long distances to access them. Therefore, most residents have no choice but to use any source of water available in their vicinity regardless of their knowledge of the risks associated. Thus, schistosomiasis control efforts should include health education and provision of adequate accessible safe water sources.

Overall, the study participants perceived that the most at risk group are SAC followed by PSAC. This observation is corroborated by studies conducted elsewhere [41]. Among the few participants who perceived women to be a high-risk group were caregivers of infected PSAC [29]. Their perception may have resulted in them not expecting their PSAC to be at risk of infection when they are playing at water contact sites, thus predisposing PSAC to schistosomiasis infection. This substantiates the need of comprehensive health education for different risk groups for infection.

Educated women had a significantly higher knowledge that haematuria is a symptom of schistosomiasis. They were also more likely to rely solely on safe water for domestic purposes compared to uneducated respondents. This strongly indicates that education is important for the community to acquire knowledge of the disease and change their behaviour to reduce the risk of acquiring infection [42, 43].

Some of the women in the ≥ 30 years age group confirmed that they were part of the previous FGS studies [17, 18], indicating that they later fell pregnant after successful treatment that resolved granulomatous lesions associated with FGS, which they thought were a cause of infertility. Their involvement in the previous studies might have resulted in them having a significantly higher knowledge of the complications of chronic schistosomiasis, compared to those < 30 years of age.

The presence and use of toilets was significantly lower in African apostolic than non-apostolic followers indicating that religion can have an impact on individual practices. Interestingly they were more likely to use safe bathing water than the non-apostolic followers. African apostolic followers are reserved, such that they shun bathing in the open area at rivers or other unprotected water bodies. Our study showed that of the 61 women who were *S. haematobium*-positive, 43 (70.5%) had toilets at home. This means that they are still being infected due to water contact, but they may not be contaminating water sources with excreta and thus infecting snails. Nevertheless, these results show that the provision of toilets alone is not adequate for elimination of indiscriminate disposal of excreta; health education emphasizing the importance and proper use of toilets for schistosomiasis control is of essence [44].

The use of unsafe water for water contact activities puts individuals directly at risk of being infected [21, 28]. We previously reported that women who allowed their child to assist in watering their garden had a significantly higher number of infected PSAC under their care while women with schistosomiasis infections and caregivers of infected PSAC were less likely to use safe bathing water [29].

Multivariate analysis has shown that the odds of using unsafe bathing water were different among the communities. The practice of using unprotected water sources was also associated with having been infected before. This shows that unsafe water sources predispose individuals to schistosomiasis infection. However, the difference in water use among the geographically close communities contributes to small-scale spatial heterogeneity in schistosomiasis transmission [45]. Previously, we reported a significant difference in schistosomiasis infection among these communities [34].

Although the study results have given an insight into the KPP of rural women in Zimbabwe, the country has diverse cultural beliefs and socio-demographic characteristics due to the existence of different tribes. Thus, KPP regarding schistosomiasis might differ by tribe or geographical area. The endemicity of schistosomiasis also varies across the country [5]. This calls for more studies in other tribes in different geographical settings with different levels of endemicity to inform targeted schistosomiasis control strategies.

## Conclusions

The present study has demonstrated that besides the high rate of schistosomiasis awareness among the respondents, some women had misconceptions about the mode of transmission, preventive measures of schistosomiasis, and poor water contact practices predisposing themselves and their PSAC to schistosomiasis infection. Development of appropriate health education tools for the community to improve their knowledge about schistosomiasis, provision of safe water and sanitary facilities to the communities and treatment will greatly improve the health and livelihoods of the communities by curtailing the transmission and morbidity caused by the disease. The findings demonstrate urgent need of integrated control programmes with a focus on health education as a strategic pillar for community behaviour change that will enable interruption of schistosomiasis transmission.

## Supplementary information

**Additional file 1: Table S1.** Association of knowledge, perceptions, and practices of the women regarding schistosomiasis with their community of residence.

## Data Availability

Data supporting the conclusions of this article are included within the article and its additional file. The datasets analysed during the present study are available from the corresponding author on reasonable request.

## Abbreviations

FGS: female genital schistosomiasis
KPP: knowledge, perceptions and practices
MDA: mass drug administration
PSAC: preschool-aged children
SAC: school-aged children
WASH: water, sanitation and hygiene
WHA: World Health Assembly
WHO: World Health Organization
